# New insights into the phenotypic spectrum of 14q22q23 deletions: a case report and literature review

**DOI:** 10.1186/s12920-018-0405-3

**Published:** 2018-09-29

**Authors:** Anna Pichiecchio, Giovanni Vitale, Camilla Caporali, Cecilia Parazzini, Donatella Milani, Maria Paola Recalcati, Laura D’Amico, Sabrina Signorini, Umberto Balottin, Stefano Bastianello

**Affiliations:** 1 Department of Neuroradiology, IRCCS Mondino Foundation, via Mondino 2, 27100 Pavia, Italy; 2 University of Pavia, Corso Strada Nuova 65, 27100 Pavia, Italy; 3 Department of Pediatric Radiology and Neuroradiology, Children’s Hospital “V. Buzzi”, via Lodovico Castelvetro 32, 20154 Milan, Italy; 4 Medical Genetics Unit, Fondazione IRCCS Ca’ Granda Ospedale Maggiore Policlinico, via Francesco Sforza 35, 20122 Milan, Italy; 5 Medical Cytogenetics Laboratory, IRCCS Istituto Auxologico Italiano, Milan, Italy; 6 Child Neuropsychiatry Unit, IRCCS Mondino Foundation, via Mondino 2, 27100 Pavia, Italy

**Keywords:** *OTX2*, MRI, Microphthalmia, Anophthalmia, Pituitary, Cerebellum

## Abstract

**Background:**

Mutations occurring in the *orthodenticle homeobox 2* gene (*OTX2*) are responsible for a rare genetic syndrome, characterized mainly by microphthalmia/anophthalmia associated with extra-ocular defects such as brain malformations, pituitary abnormalities, short stature and intellectual disability. To date, the spectrum of radiological features observed in patients with *OTX2* mutations has never been summarized.

**Case presentation:**

In this report, we describe a case of large microdeletion encompassing OTX2 but not BMP4 presenting with a syndromic anophthalmia with corpus callosum hypoplasia, pituitary gland hypoplasia and vermian hypoplasia.

**Conclusion:**

Our case report provides an illustration of the neuroradiological spectrum in a case of *OTX2*-related syndrome and the first radiological evidence of 14q22.2q23.1 deletion associated posterior cranial fossa anomalies.

## Background

The *orthodenticle homeobox 2* gene (*OTX2*, OMIM #600037) encodes a member of the bicoid subfamily of homeodomain-containing transcription factors, and it plays a crucial role in brain, pituitary gland, sensory organ and craniofacial development. More specifically, it is involved in several processes, which include: forebrain induction and specification, pituitary and GnRH neuronal system development eye formation (playing a major role in retinal pigment epithelium specification) and migration of neural crest cells from the hindbrain (which leads to the development of the maxillary and mandibular prominences) [1]. Furthermore, in the developing brain of the mouse embryo, it influences the activity of the isthmic organizer (midbrain-hindbrain boundary) through its expression in the rostral-medial ends of the cerebellar primordia (vermis-forming epithelium) [1].

Mutations in *OTX2* exhibit incomplete penetrance and broad extra and intrafamilial phenotypic variability [2, 3]. The major phenotype reported in patients with *OTX2* mutations consists of isolated or syndromic microphthalmia/anophthalmia, possibly associated with extra-ocular defects such as brain malformations, pituitary abnormalities, short stature and intellectual disability [4]. Three distinct syndromic diseases are linked to haploinsufficiency of *OTX2*, namely combined pituitary hormone deficiency 6 (CPHD6, OMIM #613986), syndromic microphthalmia 5 (MCOPS5, OMIM #610125) and otocephaly/agnathia complex [5]. *OTX2* mutations are the second most common genetic cause of microphthalmia/anophthalmia (after SOX2); furthermore, the gene is responsible for a very small proportion (less than 1%) of infantile retinal disorders, such as Leber’s congenital amaurosis [1].

The pathogenic effect is probably due to an haploinsufficiency mechanism; some cases of microdeletions encompassing *OTX2* are reported: anophthalmia/microphthalmia, other ocular defects, pituitary disfunction, anomalies of the extremities, cardiac malformations, urogenital abnormalities, are described. Regarding the extraocular involvement, the phenotypic spectrum of OTX2 mutations included structural and functional abnormalities of the pituitary gland, global developmental delay, autism, attention-deficit disorder, feeding difficulties, seizures and microcephaly other structural brain anomalies, affecting the corpus callosum and hippocampus with no clear genotype-phenotype correlations [6–9]. Also large deletions encompassing OTX2 involving also *BMP4* were previously described associated with syndromic anophtalmia phenotype including microcephaly, sensorineural deafness, abnormalities of extremities, cryptorchidism, partial callosal agenesis, cerebellar and pituitary abnormalities, and developmental delay [10].

To the best of our knowledge, we here report the first case of posterior fossa involvement in a patient with a microdeletion encompassing the *OTX2* gene, and also review the radiological findings described in literature reports of *OTX2* mutations and deletions.

## Case presentation

The patient here described is the first child of Caucasian healthy non-consanguineous parents, born at the 35th week of gestation by natural delivery following premature rupture of the membranes. The pregnancy was otherwise unremarkable. He has an older maternal half-sister, and his mother previously suffered a miscarriage at the 6th week of gestation.

**Fig. 1:**
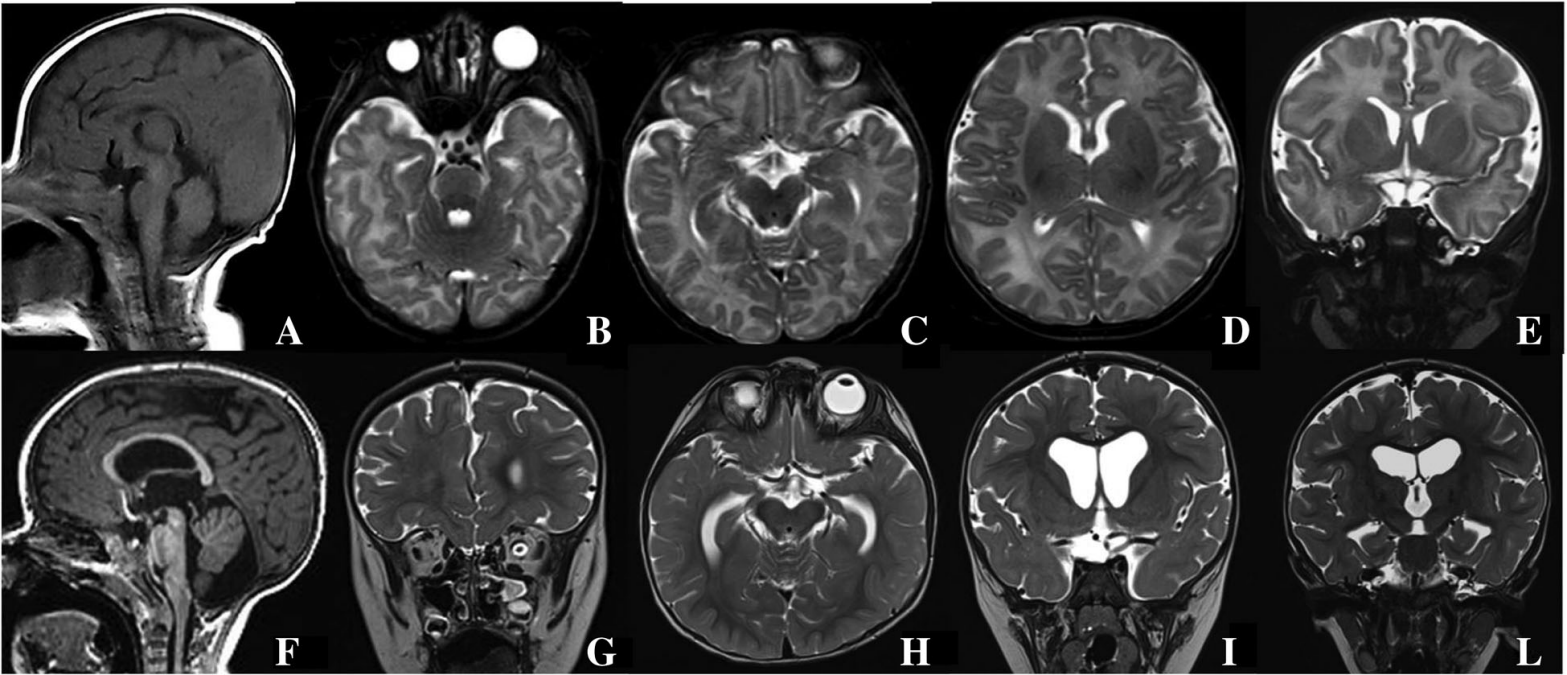
Brain MRI at birth and at the age of 18 months. Brain MRI at birth (**a**-**e**): sagittal T1-weighted spin echo (SE) (**a**), axial T2-weighted turbo spin echo (TSE) (**b**-**d**), coronal T2-weighted TSE (**e**). The examination shows right microphthalmia (**b**) and agenesis of the right optic nerve and hemi-chiasm (**a**-**c**), normal pituitary gland and stalk (**a** and **e**), small cranial posterior fossa with vertical and caudal tentorial implant, and a wider-than-normal IV ventricle due to cerebellar vermis hypoplasia (**a**). No molar tooth sign is evident at the midbrain level (**c**). Follow-up brain MRI at the age of 18 months (**e**-**l**): sagittal T1-weighted SE (**e**), coronal T2-weighted TSE (**f**, **i**, **l**) and axial T2-weighted TSE (**h**). The examination confirms the eyeball, optic nerve and posterior fossa findings, and clearly displays slight vermian dysmorphism and a wide communication between the IV ventricle and the basal cisterns (**f**), with regular superior cerebellar peduncles (**f**), corpus callosum hypoplasia (**f**), ventricular enlargement (**i** and **l**), incomplete hippocampal inversion (**l**) and pituitary gland hypoplasia (**f** and **i**)

**Fig. 2:**
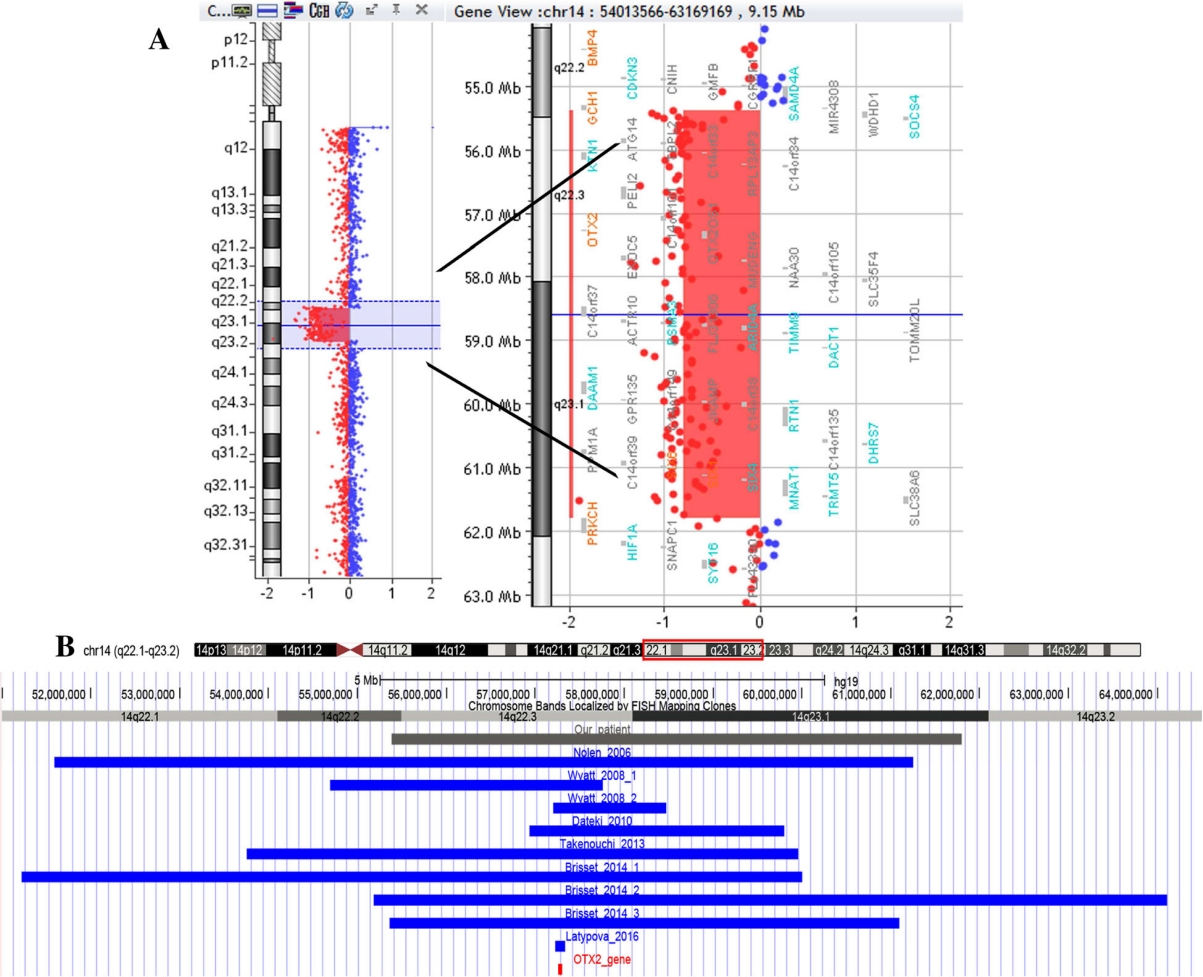
Array-CGH results and 14q22.1-q23.2 map. **a** Array-CGH profile of chromosome 14 and enlargement of the region involved in the deletion (SurePrint G3 Human CGH Microarray kit 8x60K, Agilent). **b** Chromosome 14 ideogram and physical map of the 14q22.1-q23.2 region (nucleotides 51,000,000–64,500,000, corresponding to the red box highlighted on the ideogram; UCSC Genome Browser, GRCh37/hg19): gray bar indicates the genomic region involved in the deletion of the present case; blue bars delineate the genomic regions involved in deletion reported in literature and reviewed in the present work; OTX2 gene is indicated as a red bar

At birth, the child presented with enophthalmia with right blepharophimosis, cryptorchidism and scrotal hypoplasia; auxological parameters were normal. Echocardiography and a complete abdomen ultrasound examination gave normal findings. Brain and orbital magnetic resonance imaging (MRI) (Fig. 1) showed a complex set of malformations: right microphthalmia and homolateral agenesis of the optic nerve and hemi-chiasm, a small posterior fossa with more vertical and caudal tentorial implant, and a wider-than-normal IV ventricle due to cerebellar vermis hypoplasia. The pituitary gland was normal. Blood samples were taken for array-CGH analysis (patient and parents) and molecular analysis of the microphthalmia-associated genes (*SOX2, GDF6, PAX6, SHH, RAX, OTX, VSX2*). The array-CGH analysis was performed according to standard protocols, using an oligonucleotide array with an average resolution of 130 Kb. The analysis showed a de novo 6,41 Mb deletion at 14q22.2-q23.1 (55386907–61,795,829, NCBI Build 37 - hg19, February 2009), involving 43 genes including OTX2 and other 7 genes reported as disease causing in OMIM database (Fig. 2). Molecular analysis revealed the genomic variant c.1271C > T (p.Pro424Leu) in the *SHH* gene, in heterozygosis. To date, this variant, maternally inherited, lacks clear pathogenic significance: since a genetic cause of microphthalmy had already been found and considering the mother showed no clinical or neuroradiological signs, the laboratory signaled it as probably not pathogenic.

The child underwent right eye enucleation and replacement with an ocular prosthesis. At 2 months of age, left choanal atresia was diagnosed by nasal endoscopy performed due to breathing difficulties. By the age of 9 months he was showing severe growth retardation without evident deficiency of pituitary hormones. Neurological examination revealed generalized hypotonia with preserved reflexes. He presented exodeviation and erratic movements of the left eye. Visual localization was possible only for multisensory targets placed at close range, and horizontal smooth pursuit could not be evoked without head adjustments. Visual evoked potentials showed increased latency, while electroretinography was unreliable due to the child’s uncooperativeness. No seizures were reported but EEG recording indicated poor spatiotemporal organization and generalized waveform abnormalities during sleep. At the age of 18 months, he showed severe developmental delay in terms of acquisition of the main communicative and motor milestones. He also displayed stereotypic behaviors, but was able to interact with his parents, smiling spontaneously in response to their voices and exploring their faces with his hands. Follow-up brain MRI (Fig. 1) confirmed the eyeball, optic nerve and posterior fossa findings, and revealed slight vermian dysmorphism, corpus callosum hypoplasia, a thin anterior commissure, global hemispheric white matter reduction, ventricular enlargement, a wide communication between the IV ventricle and the basal cisterns, dysmorphism of the temporal horns of the lateral ventricles due to incomplete hippocampal inversion, and pituitary gland hypoplasia (101.5 mm^3^) [11]. Pituitary hormone levels were still within the normal range, but an endocrinological follow-up remains mandatory due to the young age of the child, his severe growth retardation and the presence, from the birth, of scrotal hypoplasia.

## Discussion and conclusions

Mutations, including deletions, in *OTX2* are responsible for a broad spectrum of morphological abnormalities, associated with high phenotypic heterogeneity, proportional to the numerous pathways of cell differentiation and migration in which the gene is involved [1].

**Fig. 3:**
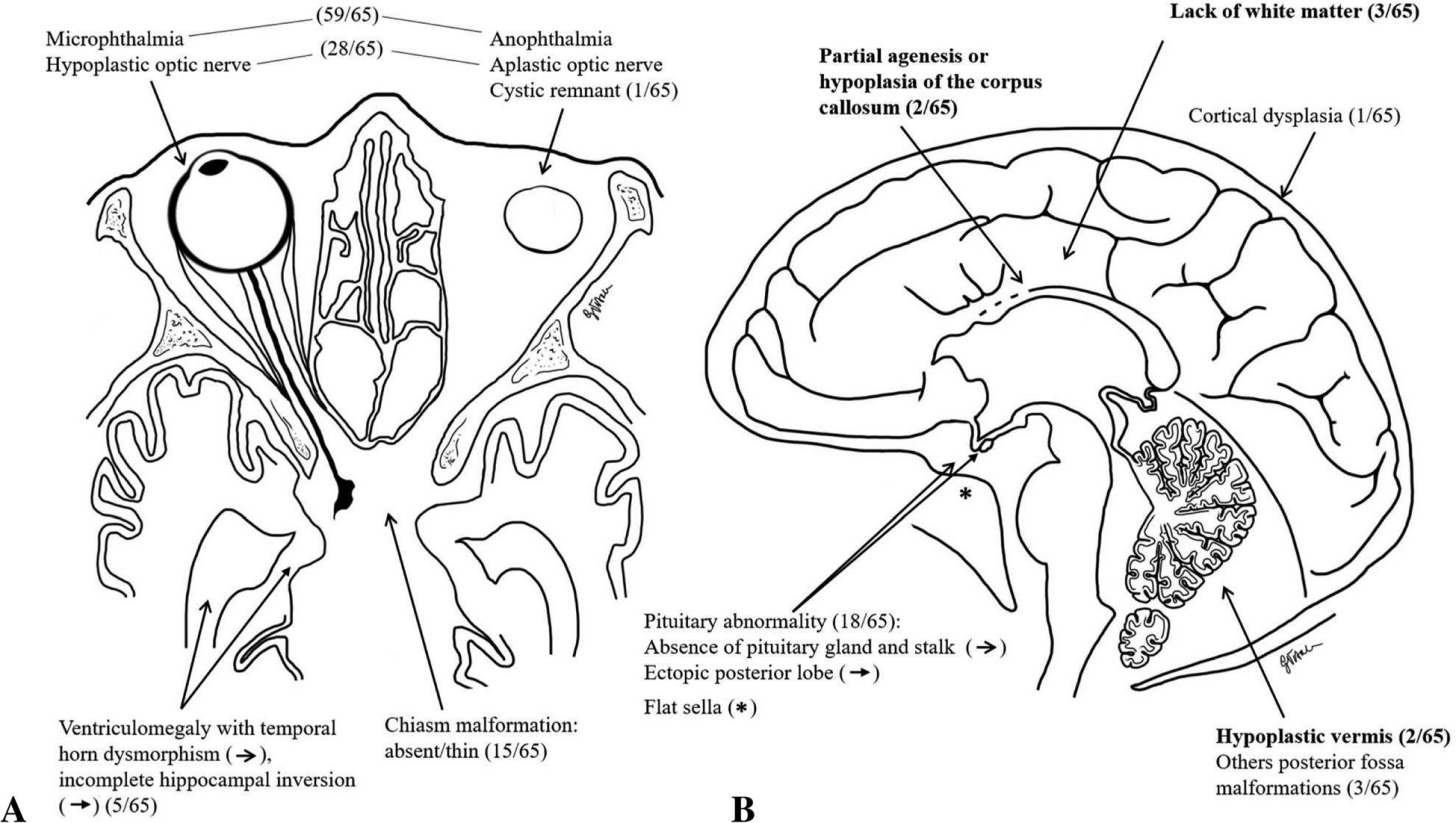
Neuroradiological findings *OTX2-*related. Graphical summary (original image, for memorization purposes) of the neuroradiological findings described in patients with *OTX2* mutations, depicted in axial (**a**) and sagittal (**b**) view. For each feature the number of patients involved is reported, referring to the total number of patients described at our knowledge (n ° 65). The alterations concerning only the patients affected by whole gene deletion are marked in bold

Brain MRI makes it possible to establish the severity of several clinically evident malformations, highlighting orbital and cerebral abnormalities that can be further subdivided into: eyeball and visual pathway dysgenesis/agenesis, pituitary malformations, and brain malformations. Table 1 summarizes the radiological findings described in available literature reports of *OTX2* mutations; these are schematically represented in Fig. 3.

**Table 1:** Summary of the radiological features associated with *OTX2* mutations reported in literature

Reference	No. of patients	Genetic mutation(s)	Proteic mutation(s)	MRI findings					
				Brain (No. of pts)	Pituitary gland (No. of pts)	Eyeball (No. of pts)	Optic nerve (No. of pts)	Chiasm (No. of pts)	Posterior fossa (No. of pts)
Bennett et al., 1991 (autopsy findings)	1	WGDel 14(q22-q23)		Geniculate bodies absent	AAL APL	bAO	bA	Absent	Small cerebellum
Elliott et al., 1993	1	WGDel 14(q22.1-q22.3)		n.a.	n.a.	bAO	n.a.	n.a.	n.a.
Lemyre et al., 1998	1	WGDel 14(q22.1-q23.2)		Cortical atrophy	HAL HPL	bAO	bA	Absent	n.a.
Ragge et al., 2005	9	c.81delC	S28PfsX23	n.a.	n.a	bMO	bH	n.a.	n.a.
		c.117_118delCC	R40GfsX47	Anterior commissure thin	Normal	bAO	bH	Thin	n.a.
		c.265C > G	R89G	Normal	Normal	bMO	bA	Absent	Normal
		c.295C > T	Q99X	Hippocampal malformation, hydrocephalus	n.a.	bAO, bilateral remnants	bA	Absent	n.a.
		c.397C > A	P133T	n.a.	n.a.	bMO,	Normal	Normal	n.a.
		c.400C > G	P134A	n.a.	n.a.	mAO	n.a.	n.a.	n.a.
		c.464insGC	S156LfsX23	Hippocampal malformation	Normal	mAO, mMO	mA, mH	n.a.	n.a.
		c.537 T > A	Y179X	n.a.	n.a.	bMO	n.a.	n.a.	n.a.
		c.537 T > A	Y179X	n.a.	Normal	bMO	bH	Thin	n.a.
Nolen et al., 2006	1	WGDel Breakpoints: 50,660,000–50,664,500 60,323,200–60,326,200 (9.6 Mb)		Ventriculomegaly, small corpus callosum, global reduction of white matter	AAL EPL	bAO	bA	Absent	n.a.
Bakrania et al., 2008	2	WGDel 14(q22.3-q23.2)		Lateral ventricles prominent Partial agenesis of corpus callosum	Abnormal	bAO	bA	Absent	Hypoplastic vermis
		14(q22.2-q23.1)		Lack of white matter	Abnormal	bAO	bA	Absent	Hypoplastic vermis
Dateki et al., 2008	1	c.402_403incC	S135LfsX2	Normal	Normal	bAO	bH	n.a.	Normal
Diaczok et al., 2008	2	c.674A > G	N225S	Normal	HAL EPL	n.a.	n.a.	n.a.	Normal
		c.674A > G	N225S	n.a.	HAL	n.a.	n.a.	n.a.	n.a.
Wyatt et al., 2008	8	c.93C > G	Y31X	n.a.	n.a.	mMO	n.a.	n.a.	n.a.
		c.106dupC	R36PfsX52	n.a.	n.a.	mMO	n.a.	n.a.	n.a.
		c.106dupC	R36PfsX52	n.a.	n.a.	mAO	n.a.	n.a.	n.a.
		c.289C > T	Q97X	n.a.	n.a.	bMO	n.a.	n.a.	n.a.
		c.289C > T	Q97X	n.a.	n.a.	Normal (coloboma)	n.a.	n.a.	n.a.
		c.371_372del AG	S125WfsX11	n.a.	n.a.	bAO	n.a.	n.a.	n.a.
		WGDel Breakpoints: 53758044–56,834,649 (3.07 Mb)		n.a.	n.a.	bMO	n.a.	n.a.	n.a.
		56,268,037–57,541,514 (1.28 Mb)		n.a.	n.a.	bAO	n.a.	n.a.	n.a.
Henderson et al., 2009	1	c.413C > G	S138X	Normal	n.a.	Normal (Leber’s congenital amaurosis)	Normal	Normal	Normal
Tajima et al.,2009	1	c.405_406insCT	S136LfsX43	Normal	HAL EPL	bAO	bA	Absent	Chiari malformation
Ashkenazi-Hoffnung a et al., 2010	1	c.270A > T	R90S	Normal	HAL EPL invisible stalk	mAO	n.a.	n.a.	Normal
Dateki et al., 2010	4	c.214_217delGC ACinsCA	A72HfsX15	Normal	n.a.	bMO	n.a.	n.a.	n.a.
		c.221_236del16	K74SfsX30	Normal	HAL, EPL	mMO, mAO	n.a.	n.a.	Normal
		c.562G > T	G188X	Normal	HAL, EPL	bMO	n.a.	n.a.	Normal
		c.562G > T	G188X	Normal	n.a.	bMO	n.a.	n.a.	Normal
Dateki et al., 2010	1	WGDel Breakpoints: 56,006,531-8,867,091 (2.9 Mb)		Normal	HAL	mMO, mAO	n.a.	n.a.	Normal
Schilter et al., 2011	5	c.136dupA	T46NfsX42	Normal	n.a.	bMO	bH	n.a.	Normal
		c.136dupA	T46NfsX42	n.a.	n.a.	bMO	bH	n.a.	n.a.
		c.313C > T	Q105X	Normal	Normal	bAO	bA	Absent	Normal
		c.456_457 delGA insAT	W152X	Normal	n.a.	mMO, mAO	bH	n.a.	Normal
		c.556_557 insTATA	S186IfsX2	Normal	HAL, EPL	bMO	bH	n.a.	Normal
Chassaing et al., 2012	Family A (7)	c.292delC	Q98NfsX11	n.a.	n.a.	MO/AO (7)	n.a.	n.a.	n.a.
	Sporadic (1)	c.106delC	R36GfsX15	n.a.	n.a.	n.a.	n.a.	n.a.	n.a.
Gorbenko Del Blanco et al., 2012	1	c.401C > G	P134R	n.a.	EPL invisible stalk	n.a.	mH	n.a.	n.a.
You et al., 2012	3	c.203G > C	R68P	Normal	Normal	mMO mAO	mH mA	n.a.	Normal
		c.203G > C	R68P	Normal	Normal	mMO	mH	n.a.	Normal
		c.203G > C	R68P	Normal	Normal	mMO	mH	n.a.	Normal
Chassaing et al., 2013	5	c.(?_-30)_(*220_?)del		Ventriculomegaly and cortical dysplasia	Normal	bAO	n.a.	n.a.	Vermian heterotopia
		c.(?_-30)_(*220_?)del		Normal	Normal	bMO and coloboma	n.a.	n.a.	Normal
		c.289C > T	R97*	Normal	Normal	mAO	n.a.	n.a.	Normal
		c.289C > T	R97*	Normal	Normal	mAO	n.a.	n.a.	Normal
		c.316delC	Q106Nfs*11	Normal	Normal	bAO	n.a.	n.a.	Normal
Patat et al., 2013^2^	1	c.289C > T	R97*	Normal	AAL APL	bMO	bA	Absent	Normal
Takenouchi et al., 2013	1	WGDel Breakpoints: 52830547–59 031284 (6.2 Mb)		Progressive white matter loss at 21 months	n.a.	bMO	n.a.	n.a.	n.a.
Brisset et al., 2014	3	WGDel Breakpoints: 50293781–59,068,634 (8.8 Mb)		n.a.	AAL EPL	bAO	bA	Absent	n.a.
		54,251,697–63,177,878 (8.9 Mb)		n.a.	AAL	bAO	bA	Absent	
		54,431,790–60,167,626 (5.8 Mb)		n.a.	AAL	bAO	bA	Absent	n.a.
Deml et al., 2016	1	c.651delC	T218Hfs*76	Normal	n.a.	bAO	Present	Present	Normal
Latypova et al., 2016	1	WGDel Breakpoints: 57166582–57,220,886 57,340,595–57,383,929 (120 Kb)		n.a.	n.a.	Normal	Normal	n.a.	n.a.
Lonero et al., 2016	1	c.402del	S135Lfs*43	Normal	EPL	mMO	mH	n.a.	Normal
Shimada et al., 2016	1	c.266G > C	R89P	Normal (lack of internal carotid artery)	HAL APL	bMO	n.a.	n.a.	Normal

*WGDel* whole gene deletion, *mMO* monolateral microphthalmia, *mAO* monolateral anophthalmia, *bMO* bilateral microphthalmia, *bAO* bilateral anophthalmia, *mH* monolateral hypoplasia, *mA* monolateral aplasia, *bH* bilateral hypoplasia, *bA* bilateral aplasia, *AAL* absent anterior lobe, *APL* absent posterior lobe, *HAL* hypoplastic anterior lobe, *HPL* hypoplastic posterior lobe, *EPL* ectopic posterior lobe, *n.a*. not available

*translation termination codon

OTX2 first expression starts within the optic vescicle, then it becomes specifically restricted to retinal pigment epithelium territory and later on is also found in the neural retina [12]. Eye development depends on the number of functional copies of *Otx,* especially of OTX2*;* embryos carrying the minimum *Otx* dosage compatible with viability show gross eye malformations such as microphthalmia or anophthalmia and agenesis of the lens. As consequence also the optic nerve, which is composed of retinal ganglion cell axons and supporting glial cells, could be affected in OTX2 mutations in the form of optic nerve hypoplasia [13].With regard to eyeball and visual pathway dysgenesis/agenesis, MRI has been shown to allow optimal characterization of microphthalmia/anophthalmia (both monolateral and bilateral), and it can reveal the presence, albeit rare, of orbital cystic remnants. Furthermore, even though *OTX2* is expressed in the optic nerve sheath, but not in the optic nerve itself, cases of optic nerve and chiasmatic hypoplasia/aplasia, as found in our patient, have been described; this finding is probably due to retrograde trans-synaptic degeneration. Interestingly, anophthalmia and microphthalmia can both be associated with optic nerve aplasia or hypoplasia.

*OTX2* mutations are also associated with variable hormonal-morphological pituitary phenotypes [1, 14]. GH is the most vulnerable pituitary hormone in *OTX2* mutations, and it can be deficient even when the gland appears normal, possibly because the gene is also involved in regulating the secretion of GnRH by the hypothalamus [14]. However, pituitary dysfunction is more commonly reported in association with developmental abnormalities of the gland, specifically anterior lobe aplasia/hypoplasia (with altered saddle conformation), ectopic/absent posterior lobe, and an invisible or interrupted stalk [14]. Our case was found to have normal pituitary function, despite showing hypoplasia of the gland, a finding which became more evident at 18 months, still in the absence of related hormonal disorders. This suggests that an MRI re-evaluation, also with 3D acquisition in doubtful cases, could provide the clinician with additional information, and therefore that the decision on whether or not it is warranted should be made independently of hormonal abnormalities.

Brain malformations in *OTX2* mutation include ventricular dilatation, partial corpus callosum agenesis, and reduced hemispheric white matter [1]. Hippocampal abnormal gyration has been described in two patients; interestingly, the hippocampus originates from the alar plate, which develops from an *OTX2*-expressing domain of the neural plate [1]. In our case we documented all these radiological findings, in particular global ventricular enlargement, diffuse white matter reduction with normal myelination, incomplete hippocampal inversion leading to dysmorphism of the temporal horns, and corpus callosum hypoplasia with a thin anterior commissure.

Moreover, our case also showed a verticalized tentorial implant bordering a small posterior fossa, and a hypoplastic and slightly dysmorphic vermis. To date, literature descriptions of malformations of the posterior cranial fossa, due to a large microdeletion encompassing both BMP4 and OTX2, consist of an old report of an autopsy finding of cerebellar hypoplasia in a fetus [15] and a more recent description of Chiari malformation [10]. The latter fails to specify the type of Chiari malformation, while the image provided deals with the pituitary findings. Moreover, two cases of vermian hypoplasia have been described, but in the presence of a concurrent *OTX2-BMP4* deletion [10]. A case of vermian heterotopia and brain cortical dysplasia has been reported, but other genetic mechanisms related to cortical development malformations were not excluded [16]. Althogh we cannot rule out a specific role of OTX2 haploinsufficiency in vermis hypoplasia, vermian involvement in cases with *OTX2* mutations could not be surprising, as it is consistent what is known about *OTX2* activity in cerebellar development. In fact, *OTX2* is expressed in the rostral-medial ends of the cerebellar primordia of the mouse embryo (the vermis-forming epithelium), suggesting that it plays a role in local neurogenesis. In support of the significance of *OTX2* in human cerebellar development, it has been demonstrated that *OTX2* acts as a repressor of myogenic and neuronal differentiation in medulloblastoma cells [1].

Microdeletions involving *OTX2* are not classically associated with cerebellar malformations. In our case, more than 20 of the genes involved in the microdeletion are expressed in cerebellum, but only three are associated with human diseases. In particular, *TMEM260* and *TRMT5* are associated with recessive diseases without cerebellar involvement, while *KIAA0586* is associated with Joubert syndrome 23 (JBTS23, MIM 616490).

Eventually, regarding phenotypic features, choanal atresia could be a misleading finding in our case report, leading the clinician to consider firstly CHARGE syndrome, due to deletion/duplication of CHD7 or SPINT2 mutations, another gene associated with developmental eye defects and choanal atresia as well as gut abnormalities. As a limit of our study, whole exome sequencing could not be performed, neither deletion/duplication analysis of CHD7 or potential coincident recessive pathogenic variants in SPINT2, but choanal atresia has been reported as associated to OTX2 mutations [8, 17]. Moreover ocular malformation of our patient characterized only by right microphthalmia, was not associated with common features of CHARGE syndrome such as coloboma, heart defects, genitourinary anomalies, ear anomalies and facial dimorphisms or with gut abnormalities typical of SPINT2 mutations.

In conclusion, our case report provides an illustration of the neuroradiological spectrum that characterizes patients with *OTX2*-related syndrome, defined by microphthalmia/anophthalmia associated with extra-ocular defects such as brain malformations, pituitary abnormalities, short stature and intellectual disability. It also provides the first radiological evidence of *OTX2* deletion with associated posterior cranial fossa anomalies.
